# Sodium–glucose cotransporter 2 inhibitor-associated erythrocytosis in type 2 diabetes: a retrospective cohort study

**DOI:** 10.1007/s00592-026-02762-w

**Published:** 2026-08-17

**Authors:** Maria Pompea Antonia Baldassarre, Federica Carrieri, Andrea Sablone, Annalisa Marotta, Paola Ranalli, Fabrizio Febo, Agostino Consoli, Marta Di Nicola, Gloria Formoso

**Affiliations:** 1 Department of Medicine and Aging Sciences, G. D’Annunzio University of Chieti-Pescara, Chieti, 66100 Italy; 2 Center for Advanced Studies and Technology (CAST), G. D’Annunzio University of Chieti-Pescara, Chieti, 66100 Italy; 3 Laboratory of Biostatistics, Department of Medical, Oral and Biotechnological Sciences, G. D’ Annunzio University of Chieti-Pescara, Chieti, 66100 Italy; 4 Hematology Unit, Department of Oncology and Hematology, Ospedale Civile “Santo Spirito”, Pescara, 65100 Italy; 5 Endocrinology and Metabolic Disease Clinic of Pescara, Pescara, 65100 Italy

**Keywords:** Erythrocytosis, Clinical Management, SGLT2 inhibitors, Type 2 diabetes

## Abstract

**Aims:**

Sodium–glucose cotransporter 2 inhibitors (SGLT2i) increase haematocrit and may cause erythrocytosis, creating uncertainty in type 2 diabetes management. Most available data derive from administrative registries or mixed, non-diabetic and/or non-European population. This study aimed to assess the onset, clinical characteristics and management outcomes of people with type 2 diabetes developing SGLT2i-associated erythrocytosis in a routine clinical setting.

**Methods:**

This retrospective, single-centre cohort study included adults with type 2 diabetes who developed at least one episode of erythrocytosis (haematocrit ≥ 48% in women, ≥ 49% in men) during the first year after initiation of SGLT2i therapy. Haemoglobin and haematocrit were collected at baseline, at erythrocytosis onset, and at 6 and 12 months with the first erythrocytosis episode defined as the “index date”. No time effect or effect modification across predictive variables was observed. Management included no intervention in 84.1%, SGLT2i discontinuation in 5.3%, and phlebotomy in 4.5%. JAK2 V617F testing was performed in 19 cases, yielding negative results.

**Results:**

Erythrocytosis was detected in 3.4% of subjects (*n* = 132 out of 3,848). The cohort was predominantly male (87.9%), with a median age of 68.4 years and diabetes duration of 10.5 years. Cardiovascular comorbidities were common. The median time from SGLT2i initiation to erythrocytosis was 11.9 months. Over 12 months, haematocrit and haemoglobin levels remained stable, with no thrombotic events.

**Conclusions:**

SGLT2i-associated erythrocytosis is an uncommon, stable, and seemingly low-risk phenomenon. However, it may trigger unnecessary interventions, including treatment discontinuation. These findings highlight a gap between the benign nature of this effect and real-world practice, supporting the need for evidence-based, multidisciplinary guidelines to preserve SGLT2i cardiorenal benefits and optimise patient management.

**Supplementary Information:**

The online version contains supplementary material available at https://doi.org/10.1007/s00592-026-02762-w.

## Background

Erythrocytosis represents an absolute increase in Haemoglobin (Hb) values from sex and race related values, requiring attention in the suspicion of malignant disorder in case of Hb levels > 16.5 g/dL in men and > 16 g/dL in women and/or haematocrit (Hct) > 49% in men and > 48% in women, according to the 2016 World Health Organization (WHO) criteria, for Polycythaemia Vera (PV) diagnosis [1]. Some risk factors have been identified in obstructive sleep apnoea syndrome (OSAS), chronic obstructive pulmonary disease (COPD), chronic kidney disease (CKD), older age, higher BMI, history of smoking, hypertension or concomitant testosterone therapy [2].

Sodium-glucose cotransporter 2 inhibitors (SGLT2i) are increasingly prescribed not only in the management of type 2 diabetes but also in cardiology and nephrology, due to robust evidence of their cardio-renal protective effects [3]. A rise in Hct is a well-known pharmacodynamic effect of SGLT2i therapy, but in a subset of individuals, erythrocytosis may develop. The underlying mechanisms may include increased erythropoietin production through hypoxia-inducible factor 2α (HIF2α) activation, modulation of iron metabolism via hepcidin suppression, and haemoconcentration [4]. Given the foreseeable increase in erythrocytosis referrals within haematology practice, formal recognition of SGLT2i-induced erythrocytosis within diagnostic algorithms has recently been proposed [5].

Reported prevalence of SGLT2i-associated erythrocytosis varies across studies, with most data derived from administrative registries or mixed populations including individuals without diabetes or non-European cohorts [5]. To date, few real-world analyses have explored the phenomenon in specialist-managed type 2 diabetes populations.

We therefore conducted a retrospective observational study in a large cohort of individuals with type 2 diabetes treated with SGLT2i to assess the occurrence, clinical characteristics, and temporal evolution of erythrocytosis at 6 and 12 months after its onset.

## Methods

### Population study

This observational, retrospective, single-centre, real-world cohort study was conducted at the Endocrinology and Metabolic Diseases Clinic of Pescara (Italy). Clinical data were collected from individuals with type 2 diabetes who started SGLT2i therapy between January 2024 and March 2025 and later developed at least one episode of erythrocytosis. Inclusion criteria were diagnosis of type 2 diabetes, ongoing treatment with any commercially available SGLT2i, and at least one episode of erythrocytosis defined as Hct value ≥ 48% in women or ≥ 49% in men. Exclusion criteria were diagnosis of type 1 diabetes and a chronic treatment with corticosteroids, erythropoietin or testosterone.

As part of standard clinical practice at our centre, all patients receiving SGLT2i therapy were counselled on the importance of adequate hydration at every follow-up visit.

Clinical and haematological data were extracted from electronic medical records routinely used in Italian diabetology clinics (Metaclinic/METEDA, San Benedetto del Tronto, Italy). Due to the observational nature of the study, all data collected as well as diagnostic and therapeutic decisions had been made according to usual clinical practice, current guidelines and individual clinical needs. Data were collected at baseline (before SGLT2i initiation), at the first occurrence of erythrocytosis, and after 6 and 12 months of follow-up considering the first erythrocytosis episode as the “index date”. For patients undergoing permanent discontinuation, phlebotomy, or switch to a different SGLT2i, an additional haematological assessment 1–3 months after the intervention was also collected, when available.

Variables included sex, age, anthropometric characteristics, diabetes duration, smoking status, macrovascular complications (peripheral arterial disease, myocardial infarction, stroke) and comorbidities such as chronic kidney disease, haematological disorders, splenomegaly, heart failure, COPD, OSAS and use of continuous positive airway pressure (CPAP), and malignancy. Concomitant therapies were recorded.

Laboratory parameters included were HbA1c, fasting glucose, creatinine and full blood count.

Information on erythrocytosis management was also collected, including SGLT2i permanent discontinuation, phlebotomy or switching to another SGLT2i.

Exclusively for the purpose of estimating the background prevalence of erythrocytosis in a population not exposed to SGLT2i, individuals with type 2 diabetes who started oral semaglutide (a glucagon like peptide-1 receptor agonist, GLP-1RA) between January 2024 and March 2025, without concomitant SGLT2i therapy, were identified from the same clinical setting and electronic medical record system, applying the same inclusion/exclusion criteria and the same 12-month observation window used for the SGLT2i cohort. No formal comparisons were performed between the two cohorts.

The study was conducted in accordance with Good Clinical Practice and the Declaration of Helsinki and received approval from the local ethics committee.

### Statistical analysis

Quantitative variables were reported as median and interquartile range (IQR) due to the non-normal distribution of the data, whereas qualitative variables were expressed as absolute frequencies (n) and percentages (%).

Longitudinal changes in Hb and Hct were evaluated using linear mixed-effects models for repeated measures, with time modelled as a within-subject factor and patient as a random effect. When subgroup comparisons were explored according to concomitant therapies (e.g., antithrombotic and antihypertensive treatments), mixed-design models were applied by including treatment group as a between-subject factor and assessing both the main effect of group and the time-by-group interaction, in addition to the within-subject time effect.

Given the retrospective nature of the study and the use of routinely collected clinical data, follow-up time was not systematically recorded for the entire population of SGLT2i-treated individuals; therefore, person-time at risk could not be reliably reconstructed. Accordingly, the frequency of erythrocytosis is reported as the proportion of patients developing at least one episode during the study period, rather than as an incidence rate per person-time, and no time-to-event analyses were performed.

Furthermore, due to the absence of a comparison group of SGLT2i-treated individuals who did not develop erythrocytosis, no analyses were conducted to identify predictors of erythrocytosis onset. Within the available dataset, analyses were therefore restricted to the evaluation of post-onset trajectories of Hb and Hct. Associations with selected clinical variables were explored using mixed-effects models and should be interpreted as descriptive and exploratory, rather than as identifying risk factors for erythrocytosis occurrence.

All tests were two-sided and a p-value < 0.05 was considered statistically significant. All statistical analyses were conducted using R software (version 4.2; http://www.r-project.org/).

## Results

Between January 2024 and March 2025, 3,848 adults with type 2 diabetes treated with an SGLT2i at the Endocrinology and Metabolic Diseases Clinic of Pescara were screened; 132 (3.4%) met the predefined Hct criteria for erythrocytosis and were included in the present study. To contextualise this finding, we assessed the occurrence of erythrocytosis in a cohort of 1,442 individuals with type 2 diabetes who started oral semaglutide without concomitant SGLT2i therapy during the same period. Only 10 subjects (0.7%) met erythrocytosis criteria, a markedly lower prevalence than observed among SGLT2i users.

The SGLT2i cohort was predominantly male with a median age of 68.4 (IQR: 62.0–77.0) years, with long-standing diabetes, managed in a single specialist diabetes centre (Table 1). At baseline (i.e. before SGLT2i initiation), Hb and Hct were 15.6 g/dL (IQR 15.0–16.2) and 46.5% (IQR 45.0–48.0), respectively (Table 1).

**Table 1:** Demographic and clinical characteristics of the cohort analysed

Variable	Category	*n* (%) or median (IQR)
Gender, *n (%)*		
	Male	116 (87.9)
	Female	16 (12.1)
Age (years), median (IQR)		68.4 (62.0–77.0)
BMI (kg/m²), median (IQR)		28.3 (26.1–33.0)
Duration of Diabetes (years), median (IQR)		10.5 (5.6–18.6)
Smoking habit, *n (%)*	Yes	28 (21.2)
	Ex	19 (14.4)
Peripheral arterial disease (PAD), *n (%)*		54 (40.9)
Acute myocardial infarction, *n (%)*		48 (36.4)
Stroke, *n (%)*		6 (4.5)
Chronic kidney disease (CKD), *n (%)*	G2	6 (11.3)
	G3A	6 (11.3)
	G3B	18 (34.0)
	G4	4 (7.5)
	G5	–
Macroalbuminuria, *n (%)*		2 (3.8)
Haematological diseases, *n (%)*		3 (2.3)
Splenomegaly, *n (%)*		–
Heart failure, *n (%)*	NYHA1	6 (4.5)
	NYHA2	10 (7.6)
	NYHA3	6 (4.5)
	NYHA4	1 (0.8)
COPD (Chronic Obstructive Pulmonary Disease), *n (%)*		–
Obstructive Sleep Apnoea Syndrome (OSAS), *n (%)*	Mild	8 (6.1)
	Moderate	–
	Severe	–
Continuous Positive Airway Pressure (CPAP), *n (%)*		1 (0.8)
Neoplastic disease, *n (%)*		11 (8.3)
Laboratory parameters at baseline (before SGLT2i initiation)		
Creatinine, (mg/dL), *median (IQR)*		1.02 (0.89–1.32)
Glomerular Filtration Rate (GFR, mL/min/1.73 m^2^), *median (IQR)*		71.6 (53.8–84.6)
White blood cells (WBC, x10^3^/µL), *median (IQR)*		8.4 (7.3–9.3)
Red blood cells (RBC, x10^6^/µL), *median (IQR)*		5.2 (5.0–5.5)
Haematocrit (Hct, %), *median (IQR)*		46.50 (45.0–48.0)
Mean corpuscular volume (MCV, fL), *median (IQR)*		89.7 (87.0–92.8)
Platelets (PLT, x10^3^/µL), *median (IQR)*		219.0 (183.0–246.7)
Haemoglobin (Hb, g/dL), *median (IQR)*		15.6 (15.0–16.2)
Glycated haemoglobin (HbA1c, *mmol/mol*), *median (IQR)*		60 (49–74)
Glycated haemoglobin (HbA1c, %), *median (IQR)*		7.6 (6.6–8.9)
Blood glucose, (mg/dL), *median (IQR)*		153.5 (129.0–193.0)
Blood glucose, (mmol/L), *median (IQR)*		8.5 (7.2–10.7)
Laboratory parameters at new-onset of erythrocytosis (Index Date)		
Creatinine, (mg/dL), *median (IQR)*		1.0 (0.9–1.3)
Glomerular filtration rate (GFR, mL/min/1.73 m^2^), *median (IQR)*		75.8 (53.6–86.3)
White blood cells (WBC, x10^3^/µL), *median (IQR)*		8.2 (7.0–9.2)
Red blood cells (RBC, x10^6^/µL), *median (IQR)*		5.6 (5.3–5.8)
Haematocrit (Hct, %), *median (IQR)*		50.0 (50.0–52.0)
Mean corpuscular volume (MCV, fL), *median (IQR)*		90.0 (86.8–93.0)
Platelets (PLT, x10^3^/µL), *median (IQR)*		208.0 (170.0–243.0)
Haemoglobin (Hb, g/dL), *median (IQR)*		16.7 (16.4–17.2)
Glycated haemoglobin (HbA1c, *mmol/mol*), *median (IQR)*		54.0 (46.0–61.0)
Glycated haemoglobin (HbA1c, %), *median (IQR)*		7.1 (6.4–7.7)
Blood glucose, (mg/dL), *median (IQR)*		135.0 (110.0–163.0)
Blood glucose, (mmol/L), *median (IQR)*		7.5 (6.1–9.0)
Concomitant medications at new-onset of erythrocytosis (index date)		
Metformin, *n (%)*		87 (65.9)
Glucagon-Like Peptide-1 Receptor Agonists (GLP1-RA), *n (%)*		39 (29.5)
Pioglitazone, *n (%)*		–
Dipeptidyl peptidase-4 inhibitors (DPP-4 inhibitors), *n (%)*		10 (7.6)
Basal insulin, *n (%)*		45 (34.1)
Rapid-acting insulin, *n (%)*		29 (22.0)
Type of SGLT2 inhibitor, *n (%)*	Dapagliflozin	60 (45.5)
	Empagliflozin	45 (34.1)
	Canagliflozin	16 (12.1)
	Ertugliflozin	11 (8.3)
Diuretics, *n (%)*	Thiazides	22 (16.7)
	Loop diuretics	26 (19.7)
	Potassium-sparing diuretics	–
Management of erythrocytosis		
Time to erythrocytosis diagnosis (months), *median (IQR)*		11.9 (3.6–29.2)
Treatment for erythrocytosis, *n (%)*	None	111 (84.1)
	Permanent discontinuation	7 (5.3)
	Phlebotomy	6 (4.5)
	Switch to other SGLT2 inhibitor	8 (6.1)
JAK2 mutations, *n (%)*	Yes	–
	No	19 (14.4)
	Not evaluated	113 (85.6)

As summarised in Table 1, the cohort showed cardiovascular and renal comorbidities, including peripheral arterial disease (PAD), previous acute myocardial infarction and heart failure, with CKD stages G2–G4 frequently represented, and traditional cardiopulmonary risk factors, including current or former smoking and obstructive sleep apnoea syndrome (OSAS). None of the individuals analysed presented a diagnosis of COPD. Dapagliflozin was the most prescribed SGLT2i, followed by empagliflozin and, to a lesser extent, canagliflozin and ertugliflozin.

The median time from SGLT2i initiation to erythrocytosis diagnosis was 11.9 months. In most individuals, erythrocytosis did not prompt any specific intervention, while only 7 (5.3%) cases underwent SGLT2i permanent discontinuation, 6 (4.5%) required phlebotomy, and 8 (6.1%) were switched to a different SGLT2i. No cases of temporary discontinuation were recorded.

Exploratory stratified analyses by management strategy showed that patients undergoing permanent discontinuation had Hct/Hb values falling below the diagnostic threshold within 1–3 months, accompanied by a rise in HbA1c (from 7.8% at onset to 8.5% post-discontinuation), while those undergoing phlebotomy showed an even more pronounced Hct/Hb decline, returning to levels comparable to the pre-treatment baseline. Switching to a different SGLT2i did not alter the Hct/Hb trajectory compared with no intervention (Supplementary Tables S2, S3). To explore inter-individual variability in erythrocytosis severity, patients were stratified by Hct > 54% at onset (*n* = 14, 10.6%), the threshold used by haematology for phlebotomy in this setting, versus Hct ≤ 54% (Supplementary Table S4). At erythrocytosis onset, the Hct > 54% subgroup had a higher prevalence of peripheral arterial disease (71.4% vs. 38.1%), a longer time from SGLT2i initiation to erythrocytosis diagnosis (32.5 vs. 11.1 months), and more frequently underwent phlebotomy or JAK2 mutation testing. Diabetes duration was longer in this subgroup (13.0 vs. 10.0 years), approaching statistical significance (*p* = 0.055). No differences were observed in baseline (pre-SGLT2i) Hct/Hb, age, BMI, or other cardiorenal comorbidities.

Testing for the JAK2 V617F mutation was performed in a minority (14.4%) and was always negative.

**Fig. 1:**
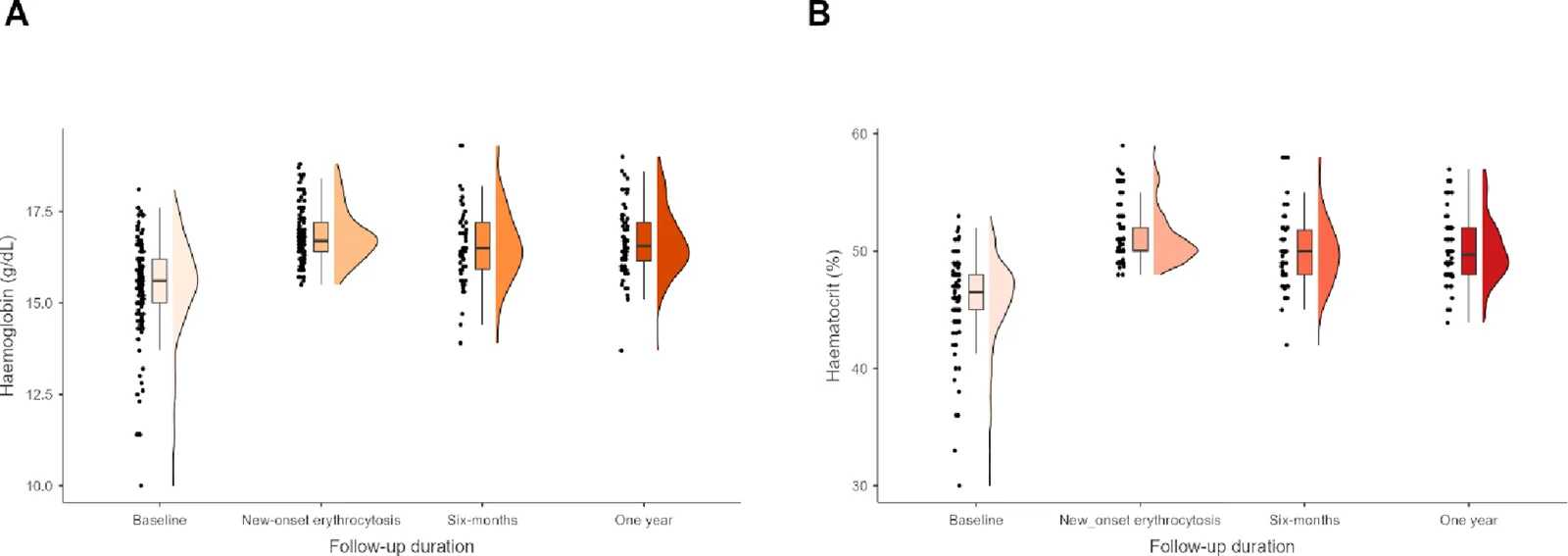
Raincloud plots illustrating longitudinal distribution of haemoglobin and haematocrit across follow-up. **A** Haemoglobin (g/dL) at baseline, new-onset erythrocytosis, 6 months, and 1 year. **B** Haematocrit (%) at the same time points. Cloud plots show the kernel-density distribution; the box indicates the median and interquartile range (IQR), whiskers extend to 1.5 × IQR, and jittered dots represent individual patient measurements at each visit. Group differences over time were assessed using a linear mixed-effects model for repeated measures (overall time effect not significant). However, haemoglobin and haematocrit were significantly higher at new-onset erythrocytosis than at baseline (*p* < 0.001), with no significant differences at subsequent time points

Hb and Hct values were significantly higher at the time of new-onset erythrocytosis compared with baseline (*p* < 0.001), as illustrated in Fig. 1. Over the subsequent 12 months of follow-up, both parameters remained overall stable, with no statistically significant changes over time. Consistently, Table 2 shows that Hb values remained stable at 6 months and 12 months across all explored strata with no significant time effect and no evidence of effect modification of all the predictive variables considered (time and time×group p-values > 0.05 throughout).

**Table 2:** Changes in haemoglobin (Hb) over time according to major cardiovascular concomitant therapies

HAEMOGLOBIN (Hb)	New-onset erythrocytosis		Six-months after erythrocytosis onset		One year after erythrocytosis onset		*p* value		
	Median	IQR	Median	IQR	Median	IQR	Time	Group	Interaction
High	16.8	16.6–17.2	16.5	15.6–17.7	16.4	16.2–17.9	***0.046***	0.201	0.120
Low	16.7	16.3–17.2	16.4	16.0-17.2	16.6	16.1–17.1			
Low-dose aspirin therapy									
Yes	16.8	16.4–17.3	16.3	15.6–17.0	16.6	15.9–17.2	0.223	0.509	0.195
No	16.7	16.3–17.0	16.5	16.2–17.2	16.6	16.2–17.0			
Anticoagulant therapy									
Yes	16.3	16.0-16.8	16.9	16.5–17.3	16.7	16.3–17.0	0.925	0.707	0.844
No	16.8	16.4–17.2	16.4	15.9–17.2	16.5	16.2–17.2			
Angiotensin II receptor blockers (ARB) therapy									
Yes	16.9	16.4–17.3	16.3	15.5–17.0	16.7	16.1–17.0	0.121	0.224	0.127
No	16.7	16.4–17.0	16.5	16.1–17.2	16.4	16.1–17.3			
β-adrenergic blocker (BB) therapy									
Yes	16.6	16.4–17.3	16.2	15.5–17.5	16.7	15.9–17.2	0.191	0.769	0.419
No	16.8	16.4–17.1	16.5	16.3–17.0	16.4	16.2–17.1			
Angiotensin-converting enzyme inhibitor (ACEi) therapy									
Yes	16.7	16.4–17.1	16.5	16.3–17.2	16.5	16.2–18.1	0.296	0.072	0.204
No	16.7	16.4–17.3	16.4	15.8–17.1	16.6	16.0-17.1			
Calcium-channel (CC) blocker therapy									
YES	16.6	16.4–17.2	16.4	15.9–17.1	16.6	16.0-16.8	0.184	0.312	0.956
NO	16.8	16.4–17.2	16.5	15.9–17.2	16.5	16.2–17.4			
Diuretic therapy									
Loops	16.6	16.2–17.5	15.9	15.3–16.5	16.2	15.6–16.7	0.351	0.054	0.065
No	16.8	16.4–17.3	16.4	16.0-17.2	16.7	16.2–17.4			
Thiazides	16.5	16.2–16.9	17	16.6–17.7	16.5	16.2–17.0			

Hb values are reported as median and IQR at each time point stratified by Hct levels (HIGH vs. LOW) and using concomitant cardiovascular treatments (aspirin, anticoagulants, ARBs, β-blockers, ACE inhibitors, calcium-channel blockers, diuretics). For each comparison, p-values are provided for the effects of Time, Group, and Time×Group interaction

Similarly, Table 3 indicates no overall statistically significant change in Hct over time across most subgroups. Moreover, no adverse events, including thrombosis or other cardiovascular events, were observed during the follow-up period. No analyses were conducted to identify predictors of erythrocytosis occurrence. The results presented in Tables 2 and 3 refer exclusively to the evaluation of Hb and Hct trajectories after erythrocytosis onset.

**Table 3:** Changes in haematocrit (Hct) over time according to major cardiovascular concomitant therapies

Haematocrit (HCT)	New-onset erythrocytosis		Six-months after erythrocytosis onset		One year after erythrocytosis onset		*p* value		
	Median	IQR	Median	IQR	Median	IQR	Time	Group	Interaction
High	51.0	50.0–51.0	51.4	48.8–52.3	50.0	48.3–52.8	0.664	0.597	0.936
Low	50.0	49.6–52.0	50.0	48.0–51.0	49.5	48.0–51.0			
Low-dose aspirin therapy									
Yes	50.4	50.0–52.0	49.5	47.0–51.0	49.0	48.0–51.0	0.222	0.233	0.271
No	50.0	50.0–51.0	50.0	48.4–51.9	50.0	49.0–52.0			
Anticoagulant therapy									
Yes	50.0	49.0-51.8	49.0	48.0-50.4	50.5	48.3–52.0	0.603	0.723	0.378
No	50.2	50.0–52.0	50.0	48.0-51.8	49.5	48.0–51.0			
Angiotensin II receptor blockers (ARB) therapy									
Yes	50.4	50.0–52.0	49.0	47.7–51.0	50.0	48.3–51.0	0.167	0.280	0.306
No	50.0	50.0-51.1	50.0	48.0–52.0	49.0	48.0–52.0			
β-adrenergic blocker (BB) therapy									
Yes	50.6	50.0–52.0	50.0	47.0-52.3	50.0	48.0–52.0	0.170	0.650	0.621
No	50.0	49.6–51.0	50.0	48.3–51.0	49.0	48.0–51.0			
Angiotensin-converting enzyme inhibitor (ACEi) therapy									
Yes	50.0	50.0–51.0	50.0	48.5–51.8	50.0	48.9–54.5	0.335	0.092	0.462
No	50.3	50.0–52.0	49.6	48.0-51.4	49.0	48.0–51.0			
Calcium-channel (CC) blocker therapy									
Yes	50.0	50.0–51.0	50.0	48.0–51.0	49.0	48.0–51.0	0.254	0.102	0.219
No	50.9	50.0–52.0	49.8	48.0–52.0	50.0	48.9–52.0			
Diuretic therapy									
Loops	51.0	50.0–53.0	49.0	46.9–50.5	49.0	48.0–52.0	0.624	0.584	0.143
No	50.0	50.0–51.0	49.9	48.0–52.0	49.0	48.2–51.8			
Thiazides	50.0	49.0-50.8	51.0	50.5–51.5	50.0	49.5–51.0			

Hct values are reported as median and IQR at each time point stratified by Hct levels (HIGH vs. LOW) and by using concomitant cardiovascular treatments (aspirin, anticoagulants, ARBs, β-blockers, ACE inhibitors, calcium-channel blockers, diuretics). For each comparison, *p-values* are provided for the effects of Time, Group, and Time×Group interaction

## Discussion

In this real-world cohort of individuals with type 2 diabetes treated in a specialist diabetology clinic, erythrocytosis during SGLT2i therapy emerged as an uncommon but not rare finding, affecting approximately 3.4% of cases. This prevalence is in line with almost prior heterogeneous evidence [5], with the exception of higher incidence (16.9%) in a Korean cohort [6], although most previous analyses were based on administrative datasets or mixed populations with or without type 2 diabetes, and rarely focused on specialist-managed type 2 diabetes Caucasian cohorts [6–9].

The substantially lower prevalence of erythrocytosis observed among GLP-1RA users (0.7%) compared with SGLT2i users (3.4%) in our clinical practice supports a class-specific pharmacological mechanism of SGLT2i.

The observed prevalence of 3.4% likely reflects a combination of pre-existing predisposing conditions and the pharmacodynamic effects of SGLT2i, rather than the contribution of either factor alone. Given the retrospective design and the absence of a non-exposed control group, however, the relative weight of each cannot be formally disentangled. *JAK2* mutation testing was consistently negative in those cases analysed, reflecting a diagnostic approach often driven by the need to exclude PV in the absence of other suggestive features.

Most cases (84.1%) did not require specific interventions, and haematological parameters remained stable over the 6- and 12-month follow-up, suggesting a phenomenon that often plateaus. This aligns with existing literature, which describes the Hct rise as generally benign and self-limiting [9]. The median time to erythrocytosis diagnosis in our cohort (11.9 months) closely mirrors the reported time to peak Hb (~ 12 months) in large observational studies [6]. This temporal overlap suggests that erythrocytosis may represent the clinical expression of a gradual and sustained erythropoietic response, rather than an early or transient effect. This interpretation is in line with current evidence indicating that SGLT2i-induced increases in Hb are mainly driven by enhanced erythropoiesis, supported by early rises in erythropoietin and reticulocyte counts, rather than hemoconcentration, which is short-lived and does not parallel the progressive increase in Hct [3, 4, 10].

Critically, this phenomenon is not merely a side effect but could be mechanistically linked to the cardiorenal benefits of this drug class. It likely reflects a complex haemodynamic adaptation, including sustained plasma volume contraction due to a reset in sodium homeostasis, together with improved renal oxygenation. Recent studies even suggest its development may be a marker of a positive haemodynamic response to the drug, associated with a reduced risk of hospitalisation for heart failure [11]. Consequently, elevated Hct may reflect underlying therapeutic efficacy and favourable haemodynamic adaptation rather than a primary adverse effect.

A critical debate centres on the clinical significance of this Hct elevation. Contrary to primary polycythaemias, SGLT2i-associated erythrocytosis does not appear to translate into an increased thrombotic risk [12–14], as also evidenced by the absence of events in our cohort. Therefore, the isolated Hct value cannot be considered a surrogate for vascular risk in this setting; the aetiological context is paramount.

The most salient observation concerns real-world clinical management. In our centre, despite the lack of specific guidelines, 5.3% of the cohort permanently discontinued therapy, with potential repercussions for glycaemic control and cardiorenal protection, as reflected by the concurrent rise in HbA1c observed in this subgroup. Furthermore, in some cases (4.5%), phlebotomy was performed, an intervention of questionable utility in this context [15]. Switching to a different SGLT2i (6.1%) did not resolve erythrocytosis either: Hct and Hb trajectories in this subgroup closely mirrored those of patients receiving no intervention, consistent with a class-wide pharmacodynamic mechanism rather than an effect specific to the individual molecule, indicating that switching within the class should not be expected to resolve this phenomenon.

Phlebotomy, when performed, was managed by haematologists or transfusion medicine doctors following referrals from primary care, generally guided by an Hct threshold of 54% adopted from the management of non-clonal erythrocytosis [16]. This threshold was not applied uniformly across cases. A clonal cause was considered unlikely on clinical basis and ruled out after molecular tests, where performed (JAK2 V617F testing in 3 of the 6 patients, all negative); the decision to proceed with phlebotomy was then individualised, taking into account hydration status, symptoms, thrombotic risk, and comorbidities, consistent with the absence of criteria specifically developed for SGLT2i-associated erythrocytosis.

Exploratory stratification by this same 54% threshold showed that patients with Hct > 54% (*n* = 14) more frequently had peripheral arterial disease and longer SGLT2i exposure, and tended to have longer diabetes duration, and more often underwent phlebotomy or JAK2 molecular testing, than those with Hct ≤ 54% (Supplementary Table S4); given the small subgroup size, these findings are hypothesis-generating rather than confirmatory.

Phlebotomy as well as drug discontinuation, while understandable in a normative vacuum, risk unnecessary medicalisation, increased healthcare costs, and potential loss of the therapeutic benefits of SGLT2i. However, an important consideration is the potential risk of diagnostic misattribution. Although rare, ascribing erythrocytosis exclusively to SGLT2i therapy could delay or preclude the necessary comprehensive diagnostic work-up for PV in a small subset of individuals supposed to be affected by both type 2 diabetes and PV.

Optimal management of *JAK*-unmutated erythrocytosis remains undefined. Gangat and colleagues have recently proposed a therapeutic algorithm focused on rigorous optimisation of cardiovascular risk factors [5]. They recommend reserving phlebotomy for symptomatic cases (such as those presenting with fatigue, headaches, visual disturbances, cognitive fog, palpitations, dyspnea, or pain) and using low-dose aspirin or anticoagulation only in individuals with a prior arterial or venous thrombotic event [5].

We acknowledge several limitations of this study. First, its retrospective, single-centre design and the relatively small sample size limit the generalisability of the findings and preclude causal inference. In addition, only individuals who developed at least one episode of erythrocytosis were included in the analysis, reflecting the descriptive aim of the study and introducing a potential selection bias.

A major limitation is the absence of a control group of SGLT2 inhibitor–treated individuals who did not develop erythrocytosis. However, the study was not designed to investigate determinants of erythrocytosis onset, but rather to characterise its clinical presentation, management, and longitudinal evolution in a real-world specialist setting. As a consequence, the identification of independent predictors and any etiological inference are beyond the scope of the present analysis, and no conclusions can be drawn regarding factors associated with the risk of developing erythrocytosis during SGLT2 inhibitor therapy.

Stratified analyses by management strategy were based on very small subgroups (*n* = 6–8) and did not allow formal statistical comparison; these results should be regarded as exploratory and hypothesis-generating. Furthermore, the lack of systematically recorded follow-up time (person-time at risk) for the entire exposed population prevented the estimation of incidence rates and the application of time-to-event analyses. Accordingly, the observed proportion of cases should be interpreted as a descriptive measure of occurrence rather than as an incidence rate.

Additional limitations include the unavailability of erythropoietin levels in most individuals and the lack of assessment of less common mutations associated with myeloproliferative disorders (e.g., CALR and MPL), beyond JAK2.

Future studies based on prospective cohort designs or administrative datasets with complete longitudinal follow-up and appropriate comparison groups are needed to provide robust incidence estimates, identify predictors of erythrocytosis onset, and better characterise its temporal dynamics.

However, this study captures what actually occurs in real-world clinical practice within a specialist diabetology setting and provides a diabetologist’s perspective on a topic that has been predominantly discussed from a haematological standpoint.

In conclusion, our data support the notion that, in type 2 diabetes, SGLT2i-induced erythrocytosis is predominantly a benign side effect, yet in clinical practice it can trigger inappropriate diagnostic and therapeutic pathways. There is an urgent need to develop shared, preferably multidisciplinary (involving diabetologists and haematologists), management algorithms. Such algorithms should promote: (1) caution in discontinuing a therapy with high cardiorenal and metabolic benefit; (2) contextual clinical evaluation before initiating extensive haematological work-up and strict monitoring; (3) education for primary care physicians and specialists about this phenomenon; and (4) establishing more carefully if avoiding SGLT2i in subgroups of individuals by themselves at high risk of erythrocytosis should be considered in order to reduce the risk of thrombotic burden.

Prospective studies are required to define intervention thresholds and long-term clinical outcomes across different clinical settings involved in the management of SGLT2i therapy (e.g., cardiology and nephrology). An additional priority is to determine which subgroups with specific risk factors may be truly prone to thrombotic complications and to identify which therapeutic strategies, if any, confer the greatest clinical benefit.

## Supplementary Information

Below is the link to the electronic supplementary material.

Supplementary Material 1
