# Supplementary material for: Sodium–glucose cotransporter 2 inhibitor-associated erythrocytosis in type 2 diabetes: a retrospective cohort study
Source: Acta Diabetol. 2026 Aug 17;63(11):1923–31. doi: 10.1007/s00592-026-02762-w (PMC13690916; doi:10.1007/s00592-026-02762-w)
Supplement: Supplementary file 1 [file 592_2026_2762_MOESM1_ESM.docx]

**Supplementary material**

**Supplementary Table S1. Baseline and index-date characteristics, concomitant medications, and management according to erythrocytosis management strategy (no intervention vs. phlebotomy vs. permanent discontinuation).**

| **Variable / Category** | **None**  **(n=111)** | **Phlebotomy (n=6)** | **Discontinuation (n=7)** | **p-value** |
| --- | --- | --- | --- | --- |
| **Demographic and clinical characteristics** | | | | |
| Gender, male, n (%) | 100 (90.1) | 5 (83.3) | 4 (57.1) | 0.033 |
| Gender, female, n (%) | 11 (9.9) | 1 (16.7) | 3 (42.9) |  |
| Age (years), median (IQR) | 68.4 (62.1–77.2) | 73.4 (72.7–74.1) | 67.5 (66.3–78.3) | 0.459 |
| BMI (kg/m²), median (IQR) | 28.2 (26.2–33.0) | 32.0 (25.0–32.8) | 30.1 (28.1–34.1) | 0.869 |
| Duration of diabetes (years), median (IQR) | 10.0 (4.0–18.0) | 19.5 (13.5–21.8) | 14.0 (7.5–22.5) | 0.071 |
| Smoking habit, current, n (%) | 22 (19.8) | 4 (66.7) | 1 (14.3) | 0.047 |
| Smoking habit, former, n (%) | 17 (15.3) | 1 (16.7) | — |  |
| Peripheral arterial disease, n (%) | 44 (39.6) | 6 (100.0) | 2 (28.6) | 0.011 |
| Acute myocardial infarction, n (%) | 41 (36.9) | 5 (83.3) | 1 (14.3) | 0.031 |
| Stroke, n (%) | 3 (2.7) | 1 (16.7) | 1 (14.3) | 0.087 |
| CKD stage G2, n (%) | 23 (20.7) | — | 1 (14.3) | 0.019 |
| CKD stage G3A, n (%) | 11 (9.9) | 1 (16.7) | 1 (14.3) |  |
| CKD stage G3B, n (%) | 21 (18.9) | 1 (16.7) | 1 (14.3) |  |
| CKD stage G4, n (%) | 3 (2.7) | — | — |  |
| CKD stage G5, n (%) | 1 (0.9) | — | — |  |
| Macroalbuminuria, n (%) | 1 (0.9) | — | 2 (28.6) | <0.001 |
| Haematological diseases, n (%) | 3 (2.7) | — | — | 0.835 |
| Heart failure NYHA I, n (%) | 4 (3.6) | — | 1 (14.3) | 0.003 |
| Heart failure NYHA II, n (%) | 9 (8.1) | — | — |  |
| Heart failure NYHA III, n (%) | 5 (4.5) | — | — |  |
| Heart failure NYHA IV, n (%) | — | 1 (16.7) | — |  |
| OSAS, mild, n (%) | 5 (4.5) | 2 (33.3) | — | 0.009 |
| CPAP use, n (%) | 1 (0.9) | — | — | 0.943 |
| Neoplastic disease, n (%) | 7 (6.3) | 2 (33.3) | 2 (28.6) | 0.013 |
| **Laboratory parameters at baseline (before SGLT2i initiation)** | | | | |
| Creatinine (mg/dL), median (IQR) | 1.02 (0.88–1.32) | 1.23 (1.22–1.24) | 1.00 (0.92–1.25) | 0.522 |
| eGFR (mL/min/1.73 m²), median (IQR) | 72.1 (53.9–84.6) | 58.4 (57.1–59.8) | 56.6 (51.5–75.3) | 0.443 |
| WBC (×10³/µL), median (IQR) | 8.4 (7.2–9.3) | 7.2 (6.7–7.6) | 8.8 (8.8–9.1) | 0.377 |
| RBC (×10⁶/µL), median (IQR) | 5.2 (5.1–5.5) | 5.1 (5.1–5.1) | 5.0 (4.9–5.4) | 0.633 |
| Hct (%), median (IQR) | 47.0 (45.0–48.0) | 47.0 (46.0–47.5) | 46.0 (45.9–46.8) | 0.909 |
| MCV (fL), median (IQR) | 89.8 (87.0–92.7) | 86.3 (82.2–90.5) | 89.4 (87.7–91.1) | 0.939 |
| Platelets (×10³/µL), median (IQR) | 221.0 (190.0–248.0) | 183.0 (168.0–184.0) | 184.0 (177.0–219.5) | 0.146 |
| Hb (g/dL), median (IQR) | 15.6 (15.0–16.4) | 15.6 (14.8–16.2) | 15.4 (14.5–15.9) | 0.820 |
| HbA1c (%), median (IQR) | 7.6 (6.6–8.9) | 7.6 (7.2–8.0) | 7.7 (6.2–9.4) | 0.979 |
| Blood glucose (mg/dL), median (IQR) | 154.0 (129.0–190.0) | 134.0 (131.0–148.0) | 127.0 (113.0–172.8) | 0.570 |
| **Laboratory parameters at new-onset erythrocytosis (index date)** | | | | |
| Creatinine (mg/dL), median (IQR) | 0.98 (0.86–1.30) | 0.96 (0.81–1.29) | 1.00 (0.99–1.24) | 0.774 |
| eGFR (mL/min/1.73 m²), median (IQR) | 76.2 (53.9–86.3) | 74.8 (58.3–81.4) | 54.4 (53.5–55.3) | 0.373 |
| WBC (×10³/µL), median (IQR) | 8.1 (7.0–9.1) | 6.8 (6.4–7.2) | 11.5 (10.3–11.8) | 0.035 |
| RBC (×10⁶/µL), median (IQR) | 5.5 (5.3–5.8) | 6.0 (5.7–6.0) | 5.6 (5.4–5.8) | 0.451 |
| Hct (%), median (IQR) | 50.0 (49.7–51.0) | 55.5 (52.8–56.5) | 52.0 (50.5–52.0) | 0.016 |
| MCV (fL), median (IQR) | 90.0 (87.0–93.3) | 91.0 (88.5–93.5) | 89.6 (88.0–91.2) | 0.984 |
| Platelets (×10³/µL), median (IQR) | 211.5 (170.8–250.2) | 154.0 (132.0–187.5) | 187.0 (179.8–195.0) | 0.118 |
| Hb (g/dL), median (IQR) | 16.7 (16.4–17.0) | 18.1 (17.5–18.1) | 16.9 (16.6–17.5) | 0.022 |
| HbA1c (%), median (IQR) | 7.0 (6.3–7.6) | 6.8 (6.4–7.3) | 7.8 (7.7–8.0) | 0.197 |
| Blood glucose (mg/dL), median (IQR) | 131.0 (108.0–162.0) | 111.0 (111.0–111.0) | 124.5 (106.8–148.0) | 0.714 |
| **Concomitant medications at index date** | | | | |
| Metformin, n (%) | 72 (64.9) | 4 (66.7) | 5 (71.4) | 0.937 |
| GLP-1 RA, n (%) | 33 (29.7) | 1 (16.7) | 4 (57.1) | 0.234 |
| DPP-4 inhibitors, n (%) | 10 (9.0) | — | — | 0.529 |
| Basal insulin, n (%) | 33 (29.7) | 4 (66.7) | 2 (28.6) | 0.163 |
| Rapid-acting insulin, n (%) | 22 (19.8) | 2 (33.3) | 1 (14.3) | 0.669 |
| **SGLT2 inhibitor type** | | | | |
| Dapagliflozin, n (%) | 52 (46.8) | 2 (33.3) | 1 (14.3) | 0.645 |
| Empagliflozin, n (%) | 36 (32.4) | 3 (50.0) | 4 (57.1) |  |
| Canagliflozin, n (%) | 14 (12.6) | 1 (16.7) | 1 (14.3) |  |
| Ertugliflozin, n (%) | 9 (8.1) | — | 1 (14.3) |  |
| **Diuretics** | | | | |
| Thiazides, n (%) | 19 (17.1) | 1 (16.7) | 1 (14.3) | 0.923 |
| Loop diuretics, n (%) | 22 (19.8) | 2 (33.3) | 1 (14.3) |  |
| **Other** | | | | |
| Time to erythrocytosis diagnosis (months), median (IQR) | 11.2 (2.6–26.9) | 29.3 (15.8–33.9) | 9.2 (6.3–16.1) | 0.176 |
| JAK2 mutation tested, negative, n (%) | 15 (13.5) | 3 (50.0) | 1 (14.3) | 0.054 |
| JAK2 mutation, not evaluated, n (%) | 96 (86.5) | 3 (50.0) | 6 (85.7) |  |

*Data are reported as n (%) or median (IQR). Percentages are calculated within each column on available data; "—" indicates zero observations. Given the limited size of the Phlebotomy (n=6) and Discontinuation (n=7) subgroups, these comparisons should be interpreted as exploratory and hypothesis-generating rather than confirmatory.*

**Supplementary Table S2. Hct, Hb, and HbA1c values (median, IQR) according to erythrocytosis management strategy, at erythrocytosis onset and at a 1–3 month window (data available in 34 [Hct/Hb] and 28 [HbA1c] patients in the no-intervention group; complete data in the phlebotomy [n=6] and discontinuation [n=7] subgroups).**

| **Variable** | **Management strategy** | **New-onset erythrocytosis (index date)** | **1–3 months post-intervention/onset** |
| --- | --- | --- | --- |
| **Hct (%)** | None | 50.00 (49.65–51.00) | 49.50 (48.00–51.00) |
|  | Phlebotomy | 55.50 (52.75–56.45) | 48.00 (45.50–49.00) |
|  | Discontinuation | 52.00 (50.50–52.00) | 46.00 (45.00–48.00) |
| **Hb (g/dL)** | None | 16.70 (16.40–17.00) | 16.45 (15.93–16.88) |
|  | Phlebotomy | 18.05 (17.48–18.10) | 15.70 (14.85–16.20) |
|  | Discontinuation | 16.90 (16.65–17.50) | 15.50 (15.30–16.40) |
| **HbA1c (%)** | None | 6.95 (6.30–7.60) | 7.00 (6.48–7.53) |
|  | Phlebotomy | 6.80 (6.40–7.28) | 7.70 (7.70–7.70) |
|  | Discontinuation | 7.80 (7.70–8.00) | 8.50 (7.75–8.65) |

*Data are reported as median (IQR). For the no-intervention ("None") group, the 1–3 months window refers to time from erythrocytosis onset; for the Phlebotomy and Discontinuation groups, it refers to time from the intervention. The 1–3 month assessment was not a protocol-defined follow-up timepoint and reflects opportunistic laboratory testing performed in clinical practice, which explains its incomplete availability in the no-intervention group. Given the very small size of the Phlebotomy (n=6) and Discontinuation (n=7) subgroups, no formal statistical testing was performed; values are presented descriptively.*

**Supplementary Table S3. Hct, Hb, and HbA1c values (median, IQR) according to switch status (switch to a different SGLT2 inhibitor vs. no switch), at erythrocytosis onset and at a 1–3 month window (data available in 30 [Hct/Hb] and 25 [HbA1c] patients in the no-switch group; complete data in the switch subgroup [n=8]).**

| **Variable** | **Switch to a different SGLT2 inhibitor** | **New-onset erythrocytosis (index date)** | **1–3 months post-switch/onset** |
| --- | --- | --- | --- |
| **Hct (%)** | Yes | 50.50 (49.75–52.00) | 51.00 (49.10–52.00) |
|  | No | 50.05 (50.00–52.00) | 49.00 (48.00–51.00) |
| **Hb (g/dL)** | Yes | 16.65 (16.28–17.07) | 16.75 (16.15–17.22) |
|  | No | 16.75 (16.40–17.20) | 16.45 (16.03–16.78) |
| **HbA1c (%)** | Yes | 8.10 (7.65–9.25) | 7.50 (7.35–8.03) |
|  | No | 7.00 (6.30–7.60) | 7.00 (6.40–7.40) |

*Data are reported as median (IQR). For the "No" (no switch) group, the 1–3 months window refers to time from erythrocytosis onset; for the "Yes" (switch) group, it refers to time from the switch. The 1–3 month assessment was not a protocol-defined follow-up timepoint and reflects opportunistic laboratory testing performed in clinical practice, which explains its incomplete availability in the no-switch group. Given the small subgroup size (switch, n=8), no formal statistical testing was performed; values are presented descriptively.*

**Supplementary Table S4. Baseline and index-date characteristics, concomitant medications and management according to erythrocytosis severity (Hct >54% vs. ≤54% at erythrocytosis onset).**

| **Variable / Category** | **Hct ≤54% (n=118)** | **Hct >54% (n=14)** | **p-value** | |
| --- | --- | --- | --- | --- |
| **Demographic and clinical characteristics** | | | | |
| Gender, male, n (%) | 102 (86.4) | 14 (100.0) | 0.216 | |
| Gender, female, n (%) | 16 (13.6) | — |  | |
| Age (years), median (IQR) | 67.5 (62.0–77.1) | 71.3 (67.5–76.5) | 0.266 | |
| BMI (kg/m²), median (IQR) | 28.3 (26.1–33.1) | 28.7 (26.5–31.4) | 0.680 | |
| Duration of diabetes (years), median (IQR) | 10.0 (4.0–18.0) | 13.0 (7.2–28.8) | 0.055 | |
| Smoking habit, current, n (%) | 24 (20.3) | 4 (28.6) | 0.764 | |
| Smoking habit, former, n (%) | 17 (14.4) | 2 (14.3) |  | |
| Peripheral arterial disease, n (%) | 45 (38.1) | 10 (71.4) | 0.022 | |
| Acute myocardial infarction, n (%) | 42 (35.6) | 6 (42.9) | 0.574 | |
| Stroke, n (%) | 5 (4.2) | 1 (7.1) | 0.497 | |
| CKD stage G2, n (%) | 23 (19.5) | 4 (28.6) | 0.594 | |
| CKD stage G3A, n (%) | 12 (10.2) | 2 (14.3) |  | |
| CKD stage G3B, n (%) | 22 (18.6) | 2 (14.3) |  | |
| CKD stage G4, n (%) | 2 (1.7) | 1 (7.1) |  | |
| CKD stage G5, n (%) | 1 (0.8) | — |  | |
| Macroalbuminuria, n (%) | 3 (2.5) | — | 1.000 | |
| Haematological diseases, n (%) | 3 (2.5) | — | 1.000 | |
| Heart failure NYHA I, n (%) | 6 (5.1) | — | 0.165 | |
| Heart failure NYHA II, n (%) | 9 (7.6) | 1 (7.1) |  | |
| Heart failure NYHA III, n (%) | 5 (4.2) | 1 (7.1) |  | |
| Heart failure NYHA IV, n (%) | — | 1 (7.1) |  | |
| OSAS, mild, n (%) | 7 (5.9) | 1 (7.1) | 1.000 | |
| CPAP use, n (%) | 1 (0.8) | — | 1.000 | |
| Neoplastic disease, n (%) | 8 (6.8) | 3 (21.4) | 0.094 | |
| **Laboratory parameters at baseline (before SGLT2i initiation)** | | | | |
| Creatinine (mg/dL), median (IQR) | 1.02 (0.88–1.32) | 1.18 (0.96–1.23) | 0.401 | |
| eGFR (mL/min/1.73 m²), median (IQR) | 72.1 (53.5–85.0) | 63.3 (57.7–82.2) | 0.570 | |
| WBC (×10³/µL), median (IQR) | 8.4 (7.0–9.3) | 9.8 (8.9–10.7) | 0.350 | |
| RBC (×10⁶/µL), median (IQR) | 5.2 (5.0–5.5) | 5.6 (5.2–5.6) | 0.292 | |
| Hct (%), median (IQR) | 46.1 (45.0–48.0) | 47.0 (46.0–49.0) | 0.222 | |
| MCV (fL), median (IQR) | 89.8 (87.0–92.7) | 86.0 (80.9–94.7) | 0.513 | |
| Platelets (×10³/µL), median (IQR) | 219.0 (183.0–250.2) | 224.5 (184.8–234.0) | 0.603 | |
| Hb (g/dL), median (IQR) | 15.6 (15.0–16.2) | 16.2 (15.1–16.7) | 0.175 | |
| HbA1c (%), median (IQR) | 7.6 (6.6–8.9) | 7.7 (7.2–8.9) | 0.785 | |
| Blood glucose (mg/dL), median (IQR) | 157.0 (132.0–200.5) | 128.0 (121.0–142.5) | 0.064 | |
| **Laboratory parameters at new-onset erythrocytosis (index date)** | | | | |
| Creatinine (mg/dL), median (IQR) | 0.99 (0.86–1.31) | 0.97 (0.95–1.23) | 0.744 | |
| eGFR (mL/min/1.73 m²), median (IQR) | 75.9 (53.5–86.4) | 74.8 (62.5–81.4) | 0.848 | |
| WBC (×10³/µL), median (IQR) | 8.4 (7.3–9.2) | 6.5 (6.3–6.7) | 0.071 | |
| RBC (×10⁶/µL), median (IQR) | 5.5 (5.3–5.8) | 5.9 (5.8–6.0) | 0.072 | |
| Hct (%), median (IQR) | 50.0 (49.2–51.0) | 56.0 (55.0–56.5) | <0.001 | |
| MCV (fL), median (IQR) | 90.0 (87.0–92.9) | 91.0 (86.0–95.5) | 0.657 | |
| Platelets (×10³/µL), median (IQR) | 208.5 (171.2–242.8) | 185.0 (162.0–266.2) | 0.791 | |
| Hb (g/dL), median (IQR) | 16.7 (16.3–17.0) | 18.2 (18.0–18.5) | <0.001 | |
| HbA1c (%), median (IQR) | 7.1 (6.4–7.8) | 7.5 (5.8–7.6) | 0.512 | |
| Blood glucose (mg/dL), median (IQR) | 141.0 (109.5–165.5) | 116.0 (111.0–122.0) | 0.145 | |
| **Concomitant medications at index date** | | | | |
| Metformin, n (%) | 81 (68.6) | 6 (42.9) | 0.074 | |
| GLP-1 RA, n (%) | 36 (30.5) | 3 (21.4) | 0.757 | |
| DPP-4 inhibitors, n (%) | 9 (7.6) | 1 (7.1) | 1.000 | |
| Basal insulin, n (%) | 39 (33.1) | 6 (42.9) | 0.553 | |
| Rapid-acting insulin, n (%) | 25 (21.2) | 4 (28.6) | 0.507 | |
| **SGLT2 inhibitor type** | | | | |
| Dapagliflozin, n (%) | 51 (43.2) | 9 (64.3) | 0.549 | |
| Empagliflozin, n (%) | 41 (34.7) | 4 (28.6) |  | |
| Canagliflozin, n (%) | 15 (12.7) | 1 (7.1) |  | |
| Ertugliflozin, n (%) | 11 (9.3) | — |  | |
| **Diuretics** | | | | |
| Thiazides, n (%) | 22 (18.6) | — | 0.185 | |
| Loop diuretics, n (%) | 22 (18.6) | 4 (28.6) |  | |
| **Management and other** | | | |  |
| Time to erythrocytosis diagnosis (months), median (IQR) | 11.1 (3.3–22.4) | 32.5 (15.8–43.0) | 0.007 | |
| Treatment: None, n (%) | 102 (86.4) | 9 (64.3) | 0.003 | |
| Treatment: Permanent discontinuation, n (%) | 6 (5.1) | 1 (7.1) |  | |
| Treatment: Phlebotomy, n (%) | 2 (1.7) | 4 (28.6) |  | |
| Switch to a different SGLT2i, n (%) | 8 (6.8) | — | 0.599 | |
| JAK2 mutation tested, negative, n (%) | 14 (11.9) | 5 (35.7) | 0.031 | |
| JAK2 mutation, not evaluated, n (%) | 104 (88.1) | 9 (64.3) |  | |

*Data are reported as n (%) or median (IQR). Percentages are calculated within each column on available data; "—" indicates zero observations. No standardised severity criteria exist for SGLT2i-associated erythrocytosis; the 54% threshold was adopted as the clinically relevant cut-off used by haematology for phlebotomy in this setting. Given the small size of the Hct >54% subgroup (n=14), these comparisons should be interpreted as exploratory and hypothesis-generating; determining the clinical or biological determinants of erythrocytosis severity was not a primary objective of the present study.*
